# RIGHTSIZING: A COLLABORATION BETWEEN PSYCHOLOGY AND EXTENSION TO HELP OLDER ADULTS DEAL WITH SENTIMENTAL CLUTTER

**DOI:** 10.1093/geroni/igae098.1700

**Published:** 2024-12-31

**Authors:** Mary Dozier, Jennifer Krafft, Pamela Turner, Diane Bales, Susan Moore, Laura Smith, Jasmine Harris-Speight, David Buys

**Affiliations:** Mississippi State University, Mississippi State, Mississippi, United States; Mississippi State University, Mississippi State, Mississippi, United States; University of Georgia, Athens, Georgia, United States; University of Georgia, Athens, Georgia, United States; University of Georgia, Athens, Georgia, United States; University of Georgia, Athens, Georgia, United States; Mississippi State University, Mississippi State, Mississippi, United States; Mississippi State University, Mississippi State, Mississippi, United States

## Abstract

Excessive clutter can create a barrier for older adults to maintain functional lives in their homes; however, interventions for hoarding are lengthy, expensive, and often unavailable, particularly for rural-dwelling older adults. Using the Rightsizing Program as an example, this presentation will highlight the synergy between Extension and academic faculty to promote aging in place across diverse states. The Rightsizing Program is a collaboration between Extension specialists at Mississippi State University (MSU) and University of Georgia (UGA) and psychology faculty at MSU with specific expertise in hoarding disorder. The program focuses on sentimental clutter, which can be particularly difficult for older adults to discard. The psychology faculty were able to integrate both their research and clinical expertise to inform program development. For example, there was an added emphasis on decreasing sense of guilt around clutter (e.g., explicitly stating that it is “okay to let go of items.”) that has been clinically observed to occur when older adults are reticent to let go of aspects of their past selves (e.g., clothes that no longer fit). The Rightsizing Program has been presented to 238 individuals and has increased access to both psychoeducation about hoarding and clutter as well as had the additional benefit of facilitating recruitment for psychological studies on hoarding on MSU campus. Partnership between Extension specialists and mental health researchers allows for a rapid dissemination of research findings, while also promoting ongoing research efforts.

